# A New Genus and Species of the Suborder Trogiomorpha (Insecta, Psocodea) from Mid-Cretaceous Amber of Myanmar †

**DOI:** 10.3390/insects13111064

**Published:** 2022-11-17

**Authors:** Xinyi Zhang, Feiyang Liang, Xingyue Liu

**Affiliations:** 1 School of Life and Health Sciences, Hunan University of Science and Technology, Xiangtan 411201, China; 2 Department of Entomology, China Agricultural University, Beijing 100193, China

**Keywords:** Burmese amber, fossil, psocids, Psocoptera

## Abstract

**Simple Summary:**

The insect order Psocodea is one of the common groups of the entomofauna from Cretaceous Burmese amber. These fossil species have shown numerous diagnostic characteristics common with the extant psocids. The psocodean suborder Trogiomorpha is considered the sister group of the other psocodean taxon. In this study, we describe a new genus that is tentatively placed into Trogiomorpha and consider it a close relative to the family Cormopsocidae. However, its familial placement is undetermined. This discovery will be a helpful contribution to understanding the ancestral characteristics of Psocodea.

**Abstract:**

We established a new genus with a new species *Brachyantennum spinosum* Liang et Liu, gen. et sp. nov. from mid-Cretaceous Burmese Kachin amber. It is tentatively placed into the suborder Trogiomorpha, based on the strong external valve, the reduced dorsal and ventral valve, and the short subgenital plate covering the basal part of the external valve. This new genus is apparently close to the family Cormopsocidae, based on the well-developed and very long hindwing Sc vein. However, its familial placement is ambiguous and it can be excluded from the established families of Trogiomorpha by the presence of the tarsal ctenidiobothria on the mid- and hindleg.

## 1. Introduction

The insect order Psocodea, an inconspicuous group, includes parasitic lice, book lice, and bark lice [1,2]. Psocodea is divided into three suborders: Trogiomorpha Roesler, 1944, Trcotomorpha Roesler, 1944, and Psocomorpha Weber, 1936 [3]. The monophyly of all the suborders has been confirmed by morphological and molecular data, and Trogiomorpha is considered as the sister group to Trcotomorpha plus Psocomorpha [4,5,6]. However, the origin of Psocodea and the divergence of these suborders are controversial in different studies because of insufficient fossil data [4,7,8]. Impression fossils of Psocodea before the Cretaceous period are extremely rare, and it is hard to gain enough morphological data from these materials [9]. Therefore, psocids from Cretaceous amber are potentially useful materials that can be used in phylogenetic dating analyses.

Recently, two trogiomorphan families were reported based on some well-preserved materials from mid-Cretaceous Burmese amber, i.e., Prionoglarididae Keny, 1930, and Cormopsocidae Yoshizawa et Lienhard, 2020 [10,11,12,13]. Prionoglaridiae is known as a “living fossil” group and is probably the most basal family of extant psocids [14]. Azar et al. [10] established the fossil genus *Palaeosiamoglaris* Azar et al., 2017, from mid-Cretaceous Burmese amber and placed it into Prionoglaridiae based on the diagnostic characteristics of its wings, especially the broad and round wings, the developed and strongly curved forewing Sc vein, and the broad ap cell. Yoshizawa and Lienhard [12] established the family Cormopsocidae based on a fossil species from mid-Cretaceous Burmese amber and estimated it to be the sister group of all other trogiomorphan taxa. The diagnostic characteristics of Cormopsocidae are as follows: antennae with numerous short flagellomeres; forewing anterior and posterior margins almost parallel; forewing Sc vein developed and arched, ending at the R vein; and the hindwing Sc vein well-developed and very long [12]. Hitherto, all members of Cormopsocidea have been found in Burmese amber, including three genera with seven species, i.e., *Cormopsocus baleoi*, Hakim, Azar and Huang, 2021, *Cormopsocus groehni* Yoshizawa and Lienhard, 2020, *Cormopsocus neli* Hakim, Azar and Huang, 2021, *Cormopsocus perantiqua* (Cockerell, 1919), *Longiglabellus edentatus* Wang, Li and Yao, 2021, *Longiglabellus pedhyalinus* Wang, Li and Yao, 2021, and *Stimulopsocus jiewenae* Liang and Liu, 2022 [11,12,13,15,16,17].

In this study, we describe a new genus and species of Trogiomorpha from mid-Cretaceous Burmese Kachin amber. It is close to the family Cormopsocidae, but its familial placement is undetermined.

## 2. Materials and Methods

The amber specimen herein studied is from the Hukawng Valley in Tanai Township, Kachin State, northern Myanmar, and it is earliest Cenomanian in age, 98.8 ± 0.62 million years, by U-Pb dating of zircons from the volcanoclastic matrix of the amber [18,19].

Observations were made by using an Olympus CX-33 (Olympus Imaging Corporation, Tokyo, Japan) light microscope. Photographs and drawings were taken by using a Sony Alpha 7II (Sony Corporation, Tokyo, Japan) digital camera attached to the Olympus CX-33. The figures were prepared with Adobe Photoshop 24.0.0 (Adobe, San Jose, CA, USA).

The morphological terminology follows Yoshizawa [20]. Abbreviations for body parts measured are: f1-fn: flagellomeres 1-n; mx2: second maxillary palp; mx4: fourth maxillary palp; ft1, ft2, ft3: first, second, and third tarsomere of foreleg; mt1, mt2, mt3: first, second, and third tarsomere of midleg; ht1, ht2, ht3: first, second, and third tarsomere of hindleg.

This manuscript has been registered in ZooBank under the number urn:lsid:zoobank.org:pub:683E3489-FE07-4E6D-9049-C28921BCAC53.

## 3. Results

Order Psocodea Hennig, 1966

Suborder Trogiomorpha Roesler, 1944

Family incertae sedis

Genus *Brachyantennum* Liang et Liu gen. nov.

Type species. *Brachyantennum spinosum* Liang et Liu, sp. nov., here designated.

Etymology. The new genus is named after the short antennae (brachy: “short” in Latin; antenn: common “antennae” in Latin). Gender neutre.

Diagnosis. Macropterous. Ocelli present; antennae very short, with 11 antennomeres, flagellomeres with secondary annulations; lacinia rob-like; mx2 and mx4 without conical sensillum, mx2 longer than mx4. Forewing: base of anterior margin with setae; veins with one row of setae; Sc well developed and forming arched, joining R near base of pterostigma; R_1_ curved, without angle; crossvein r-rs present, connecting pterostigma and Rs; ap cell broad, subtriangular; CuP and A ending closely, but not meeting on forewing margin; nodulus absent. Hindwing glabrous, Sc very long and ending on wing margin; A bifurcate, A1 curved. Leg: femora and trochanters without spines; tibiae bearing spines; tarsus three-segmented; 1st tarsus with one row of ctenidiobothria on mid- and hindleg; pretarsal claw symmetrical, with a preapical tooth. Female genitalia strongly sclerotized. Clunium simple, not fused to subgenital plate. Epiproct with short setae. Paraproct with long setae. External valves strong and broad, with several spurs on the margin.

**Figure 1:**
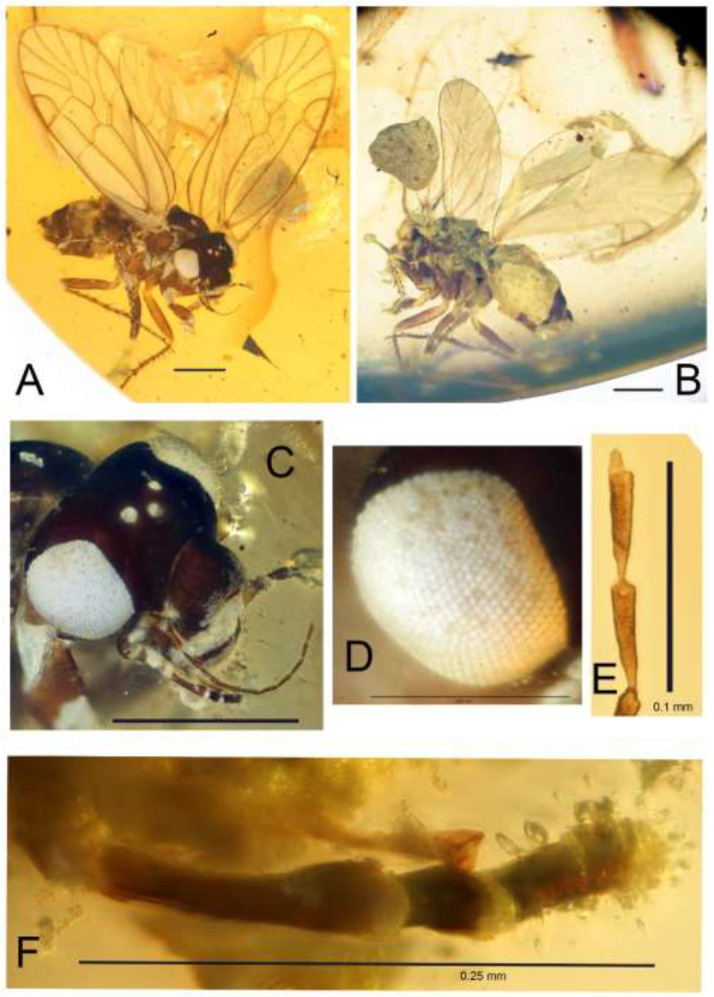
*Brachyantennum spinosum* gen. et sp. nov., holotype female. (**A**) Habitus, right lateral view, scale bar = 0.5 mm; (**B**) Habitus, left lateral view, scale bar = 0.5 mm; (**C**) Head, scale bar = 0.5 mm; (**D**) Compound eye, scale bar = 0.25 mm; (**E**) Tip of antennae, scale bar = 0.1 mm; (**F**) Lacinia and maxillary palp, scale bar = 0.5 mm.

**Figure 2:**
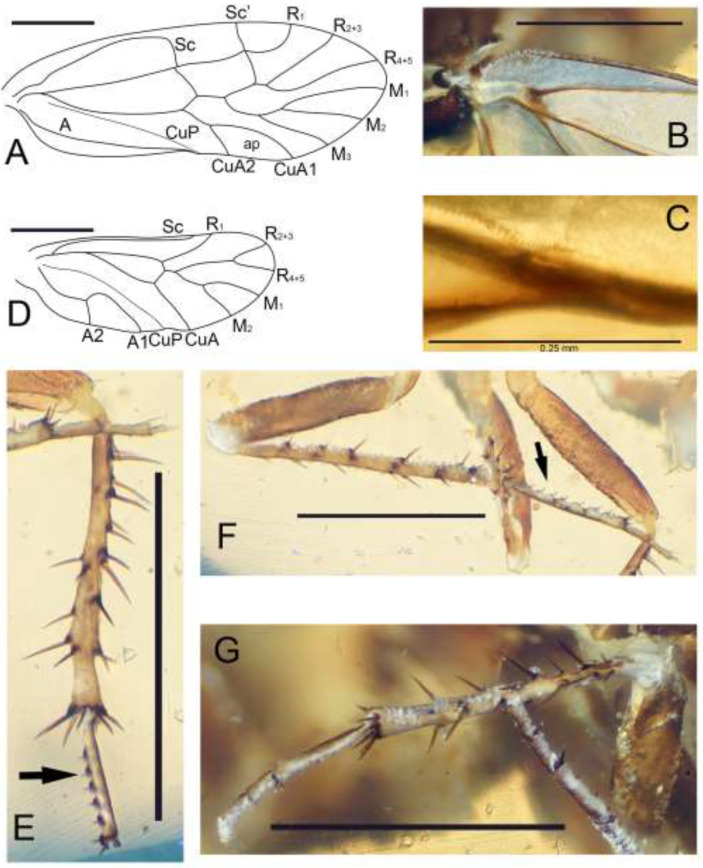
*Brachyantennum spinosum* gen. et sp. nov., holotype female. (**A**) Forewing, scale bar = 0.5 mm; (**B**) Base of forewing anterior margin, scale bar = 0.5 mm; (**C**) In-flight wing-coupling structure, scale bar = 0.25 mm; (**D**) Hindwing, scale bar = 0.5 mm; (**E**) Tibia and 1st tarsus of right midleg, arrow to ctenidiobothria, scale bar = 0.5 mm; (**F**) Left hindleg, arrow to ctenidiobothria, scale bar = 0.5 mm; (**G**) Right hindleg, scale bar = 0.5 mm. ap: areola postica.

**Figure 3:**
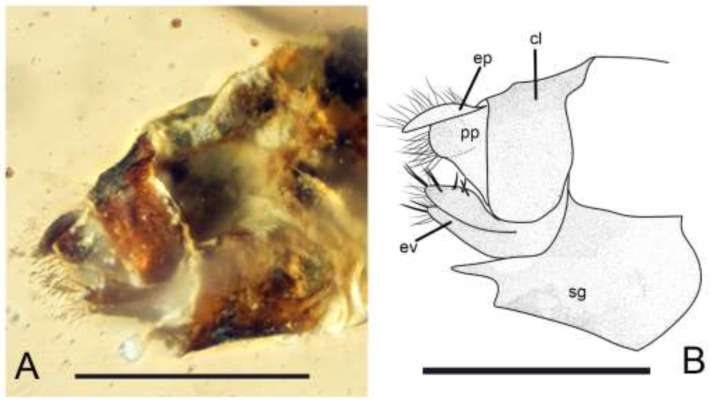
*Brachyantennum spinosum* gen. et sp. nov., holotype female. (**A**) Photograph of genitalia, scale bar = 0.5 mm; (**B**) Drawing of genitalia, scale bar = 0.5 mm. cl: clunium; ep: epiproct; pp: paraproct; ev: external valve; sg: subgenital plate.

*Brachyantennum spinosum* Liang et Liu, sp. nov., (Figure 1, Figure 2 and Figure 3).

Etymology. The new species is named after the external valve with spurs.

Type material: Holotype, CAU-BA-LFY-21003, female, deposited in the School of Life and Health Science, Hunan University of Science and Technology. The type specimens will eventually be deposited in the Entomological Museum, China Agricultural University (CAU), Beijing, China, after the authors end their careers.

Type locality and horizon: Hukawng Valley, Kachin State, northern Myanmar. Mid-Cretaceous Cenomanian period, 98.79 ± 0.62 Ma.

Diagnosis. As for the genus.

Description. Female. Body length 2.19 mm (measured from postclypeus to terminalia). f1 = f2 = f3 = 0.08 mm. Forewing length 2.59 mm, width 0.94 mm; hindwing length 1.86 mm, width 0.70 mm. ft1 = ft2 = 0.05 mm, ft3 = 0.07 mm; mt1 = 0.21 mm, mt2 = mt3 = 0.06 mm; ht1 = 0.40 mm, ht2 = ht3 = 0.09 mm.

Coloration. Head, thorax and legs dark brown. Compound eyes white, with distinct color pattern; ocelli white. Fore- and hindwing transparent. Abdomen dark brown.

Head (Figure 1). Compound eyes (Figure 1C) and ocelli (Figure 1D) bulged. Vertical suture visible, frontal suture not visible, epistomal suture distinct. Antenna filiform, with 11 antennomeres; flagellomeres short, with secondary annulations (Figure 1E). Lacinia (Figure 1F) simplified, rob-like. Maxillary palps four-segmented, without visible sensillum on any segment, mx2 longer than mx4. Labial palps two-segmented.

Thorax well preserved. Prothorax short. Meso- and metathorax robust.

Wings (Figure 2A–D) transparent, with developed venation. Forewing (Figure 2A–C): base of anterior margin (Figure 2B) with setae; veins with one row of setae; Sc well developed and forming arched, joining R near 2/3 R; R_1_ arc, without posterior angle; Rs with two branches; crossvein r-rs present, connecting pterostigma and Rs; M with three branches; CuA with two branches, ap cell broad; CuP and A ending closely, but not meeting; nodulus absent; in-flight wing-coupling structure simple. Hindwing (Figure 2D): glabrous; Sc long and ending at 2/3 distal of anterior margin; Rs and M with two branches; CuA and CuP simple; A bifurcate, A1 curved, A2 straight.

Legs (Figure 2E–G). All tibiae with spines and more than two apical spurs. Tarsi three-segmented; foreleg 1st tarsus without ctenidiobothria, midleg and hindleg 1st tarsus with one row of ctenidiobothria (Figure 2E,F); midleg and hindleg 1st tarsus much longer than foreleg 1st tarsus. Pretarsal claws symmetrical, with a preapical tooth, no pulvillus.

Abdomen. Female genitalia strongly sclerotized. Clunium simple, not fused to subgenital plate. Epiproct with short setae, paraproct with long setae. A pair of strong and broad external valves visible in lateral view, appeared out of subgenital plate, with several spurs on the tip of external valve. Subgenital plate broad and strongly sclerotized.

## 4. Discussion

Yoshizawa et al. [21] concluded that the suborder Trogiomorpha can be identified based on three morphological apomorphic features: (1) the reduced ventral and dorsal valves; (2) the well-developed external valve close to the ventral midline of the abdomen; and (3) the short subgenital plate, covering at the basal part of the external valves. Therefore, we placed the new genus *Brachyantennum* gen. nov. into Trogiomorpha based on these characteristics.

*Brachyantennum* gen. nov. is similar to the two families Cormopsocidae and Prionoglaridiae in the forewing venation, especially in the well-developed and strongly curved Sc, and the presence of a cross-vein between the pterostigma and Rs. Yoshizawa and Lienhard [12] considered these characteristics to be the ancestral conditions of Trogiomorpha. The long antennae and the round forewing with an obviously curved anterior margin are considered the apomorphic features of Prionoglarididae [12,22]. All extant members of Prionoglarididae are cave dwellers, but *Brachyantennum* gen. nov. has no cave-dwelling behavior because of the dark body coloration and the short antennae. Thus, *Brachyantennum* gen. nov. can be excluded from Prionoglarididae by the dark body coloration, the extremely short antennae, and the slightly curved anterior margin of the forewing.

*Brachyantennum* gen. nov. is closer to Cormopsocidae than Prionoglarididae, based on the well-developed and very long hindwing Sc vein, which is recognized as the most prominent characteristic of Cormopsocidae by Yoshizawa and Lienhard [12]. The hindwing Sc vein of Cormopsocidae is slightly curved and ends near the mid anterior margin of the hindwing [11,12,13,15,16]. In contrast, the base of the hindwing Sc vein of *Brachyantennum* gen. nov. is obviously curved, and the Sc vein ends near the 2/3 distal of the anterior margin of the hindwing. However, *Brachyantennum* gen. nov. is obviously larger than the members of Cormopsocidae in the body and wings. Remarkably, the presence of basal tarsal ctenidiobothria on the mid- and hindlegs is very characteristic of *Brachyantennum* gen. nov.. In the suborder Trogiomorpha, the tarsal ctenidiobothrium is generally reduced [23]. In the suborder Trocomorpha, the ctenidiobothria are only present on the basal tarsal of the hindleg in a few families, such as Amphientomoidea Enderlein, 1903, and Manicapsocidae Mockford, 1967 [23,24]. In contrast, the presence of basal tarsal ctenidiobothria on the mid- and hindlegs is generally observed in some families of the suborder Psocomorpha, such as Stenopsocidae Pearman, 1936, and Psocidae Hagen, 1865. Therefore, *Brachyantennum* gen. nov. can be excluded from the family Cormopsocidae by the tarsal ctenidiobothria.

Based on the discussion above, the placement of *Brachyantennum* gen. nov. is ambiguous. We tentatively place it into the suborder Trogiomorpha, based on the characteristics of the external valve. However, this genus cannot be assigned to any established families of Trogiomorpha. We do not propose a new family, because it shows numerous plesiomorphic characteristics based on the female individual. The discovery of male materials of this genus would provide more information to determine its placement in the order Psocodea.

## Data Availability

All data generated during this study are included in this published article. The type specimen (CAU-BA-LFY-21003) is deposited in the School of Life and Health Science, Hunan University of Science and Technology and will eventually be deposited in the Entomological Museum, China Agricultural University (CAU), Beijing, China after the authors end their careers.
